# Supplementary material for: Evaluation of Oral Mucosal Lesions Using the IllumiScan® Fluorescence Visualisation Device: Distinguishing Squamous Cell Carcinoma
Source: Int J Environ Res Public Health. 2022 Aug 21;19(16):10414. doi: 10.3390/ijerph191610414 (PMC9408154; doi:10.3390/ijerph191610414)
Supplement: Supplementary file 1 [file ijerph-19-10414-s001.zip › ijerph-1793261-supplementary.pdf]

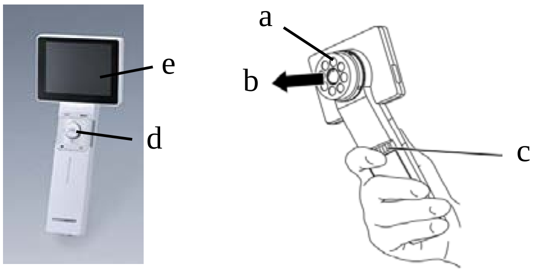

Figure S1. The IllumiScan® fluorescence visualisation device (Shoufu, Kyoto, Japan)

Eight LED ring lights (a) emit 425-nm blue light (b). The camera is focused using the dial (c), and the picture is captured using the centre button (d). The image is checked on a monitor (e). Source: IllumiScan® user manual.

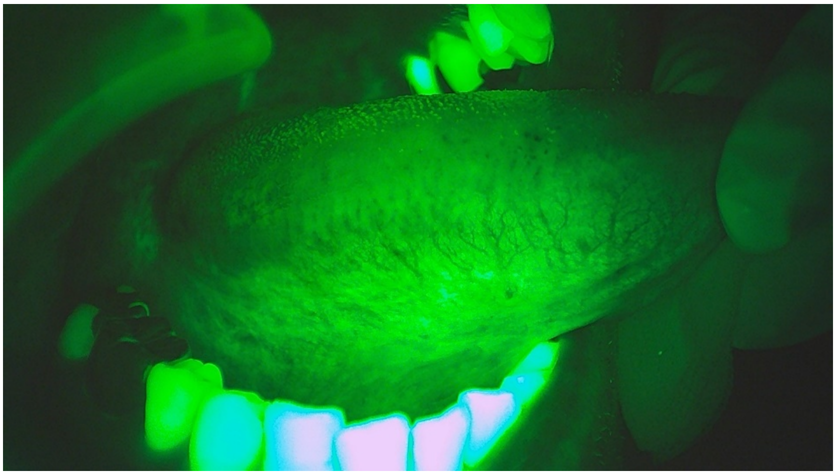

Figure S2. The right side of a participant’s tongue showing normal mucosa in the fluorescence visualisation image.

In a 55-year-old man, autofluorescence (green) of the sublingual mucosa is observed. The sublingual vein under the mucosa absorbs the irradiated light so that the blood vessel can be observed as a result of loss of fluorescence.

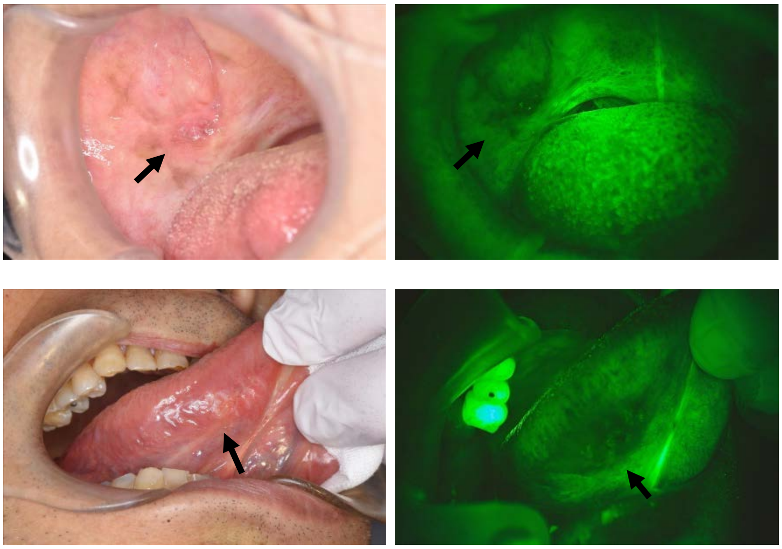

Figure S3. IllumiScan® is highly sensitive. It is easy to miss lesions in intraoral photographs, but with IllumiScan®, fluorescence loss in lesions can be clearly observed.

A) A large (8 mm) ulcer is observed on the right buccal mucosa, but it was difficult to visualise it under white light. The pathological diagnosis was squamous cell carcinoma (SCC). Clear fluorescence loss is observed in the IllumiScan® image.

B) A 21 × 10-mm mass is observed on the sublingual surface of the lower left bicuspid. The surface was erythematous, and there was induration around it. However, this was difficult to observe under white light. The pathological diagnosis was SCC. Clear fluorescence loss can be observed in the IllumiScan® image.

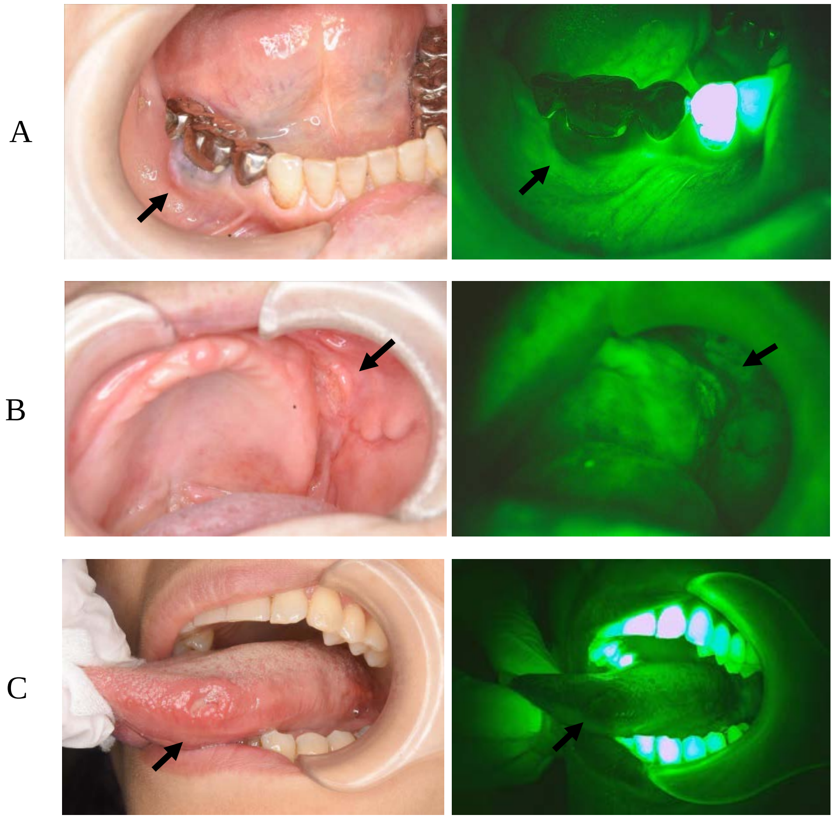

Figure S4. It is difficult to distinguish between cancer and inflammation in fluorescence visualisation images.

A) Fluorescence visualisation loss (FVL) in the gingiva of the cervical region of the lower right molar. Pathological results showed chronic mucositis.

B) FVL is seen at the left-lateral gingival border transition. Pathological results showed an irritation fibroma with ulceration.

C) FVL at the left lingual border. Pathological examination revealed an inflammatory lesion.

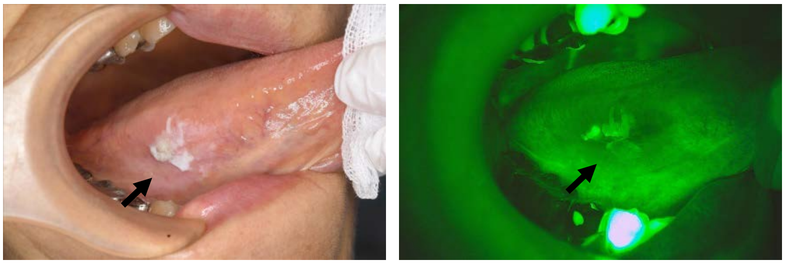

Figure S5. Enhanced intensity due to hyperkeratosis obscures the lesion.

A white lesion measuring 25 × 10 mm is visible on the right lingual margin. The lesion was a fine granular mass with a central induration of 5 × 5 mm. The pathological diagnosis was squamous cell carcinoma. Fluorescence visualisation loss in the lesion was obscured by fluorescent intensity enhancement in the keratinised area on the fluorescence visualisation image.

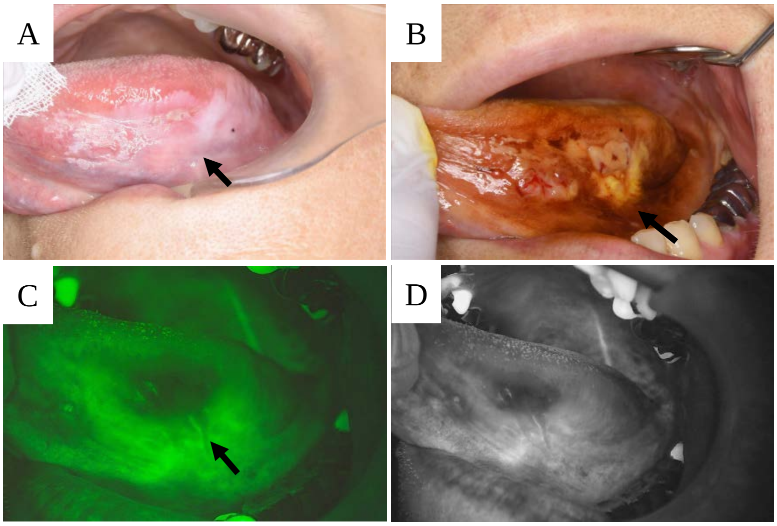

Figure S6. Determining the region of interest (ROI).

The lesion on the left tongue, with a 10-mm large ulcer and surrounding white patches was histopathologically diagnosed as squamous cell carcinoma.

A) Photograph was taken under white light. B) Lugol's staining. C) Illumiscan images. D) The ROI (solid yellow line) of the lesion was defined as the area with fluorescence visualisation loss in the black-and white image created from the Green image. Then, the intensity in the ROI was measured with ImageJ software.

Table S1. Comparison of our results with those of other facilities

Equipment	Author	Facility	Year	Lesion	Cases	Image analysis	Average area	Average FI	Average CV
IllumiScan	Morikawa[35]	Tokyo Medical and Dental University	2017	SCC	30	ImageJ	97×10^3	37.9	0.23
				OLP	25		52×10^3	61.3	0.14
				control	55		820	73.6	-
IllumiScan	Kikuta[19]	Kurume University	2018	SCC	31	ImageJ	69×10^3	93.4	0.3
				leukoplakia	9		29×10^3	146.6	0.21
				control	40		19×10^2	145.9	0.24
IllumiScan	Sugahara[33]	Tokyo Medical and Dental University	2020	SCC	7	original**	21×10^2	56	-
				control	7		21×10^2	147	-
IllumiScan ORALOOK	Morikawa[34]	Tokyo Medical and Dental University	2020	SCC,	100	ImageJ	16×10^4	54.5	0.22
				OPMD	214		34×10^3-11×10^4	62.2-85.2	0.12-0.13
				control	314		830-890	80.0-82.7	0.040-0.045
IllumiScan	Present study	Hiroshima University	2019~2021	SCC	59	ImageJ	37×10^3*	88*	0.203*
				non-SCC	131		31×10^3*	109*	0.134*
				NOM	49		85×10^3*	155*	0.135*

*median

**Numerical settings for luminance are the same as in ImageJ. CV, coefficient of variation; FI, fluorescence intensity; SCC, squamous cell carcinoma; OPMD, oral potentially malignant disorder; OLP, oral lichen planus
